# HIV-1 Group M Capsid Amino Acid Variability: Implications for Sequence Quality Control of Genotypic Resistance Testing

**DOI:** 10.3390/v15040992

**Published:** 2023-04-18

**Authors:** Kaiming Tao, Soo-Yon Rhee, Philip L. Tzou, Zachary A. Osman, Sergei L. Kosakovsky Pond, Susan P. Holmes, Robert W. Shafer

**Affiliations:** 1 Division of Infectious Diseases, Department of Medicine, Stanford University, Stanford, CA 94305, USA; 2 Institute for Genomics and Evolutionary Medicine, Temple University, Philadelphia, PA 19122, USA; 3 Department of Statistics, Stanford University, Stanford, CA 94305, USA

**Keywords:** HIV-1, capsid, lenacapavir, drug resistance, subtype, cytotoxic T lymphocytes

## Abstract

Background: With the approval of the HIV-1 capsid inhibitor, lenacapavir, capsid sequencing will be required for managing lenacapavir-experienced individuals with detectable viremia. Successful sequence interpretation will require examining new capsid sequences in the context of previously published sequence data. Methods: We analyzed published HIV-1 group M capsid sequences from 21,012 capsid-inhibitor naïve individuals to characterize amino acid variability at each position and influence of subtype and cytotoxic T lymphocyte (CTL) selection pressure. We determined the distributions of usual mutations, defined as amino acid differences from the group M consensus, with a prevalence ≥ 0.1%. Co-evolving mutations were identified using a phylogenetically-informed Bayesian graphical model method. Results: 162 (70.1%) positions had no usual mutations (45.9%) or only conservative usual mutations with a positive BLOSUM62 score (24.2%). Variability correlated independently with subtype-specific amino acid occurrence (Spearman rho = 0.83; *p* < 1 × 10^−9^) and the number of times positions were reported to contain an HLA-associated polymorphism, an indicator of CTL pressure (rho = 0.43; *p* = 0.0002). Conclusions: Knowing the distribution of usual capsid mutations is essential for sequence quality control. Comparing capsid sequences from lenacapavir-treated and lenacapavir-naïve individuals will enable the identification of additional mutations potentially associated with lenacapavir therapy.

## 1. Introduction

The HIV-1 capsid is expressed as part of the Gag polyprotein. Following the assembly and budding of viral particles from infected cells, the capsid is released by proteolytic cleavage during viral maturation. The capsid subunits assemble into a cone-shaped core composed of approximately 250 hexamers and exactly 12 pentamers. During the early stages of viral replication, the core is involved in reverse transcription, controlled disassembly, and nuclear transport of viral DNA. During the later stages of replication, the HIV-1 capsid participates in viral assembly and maturation [1,2].

Each capsid monomer consists of 231 amino acids containing an N-terminal domain (NTD) of 145 amino acids with a β-hairpin and seven α-helices, a C-terminal domain (CTD) of 81 amino acids with four α-helices, and an unstructured interdomain linking region of five residues. Intra-hexameric NTD-NTD and NTD-CTD contacts between capsid molecules stabilize individual hexamer and pentamer building blocks [3]. Inter-hexameric CTD-CTD contacts participate in dimeric and trimeric interactions to link individual hexamers and pentamers. In addition, multiple capsid motifs are involved in critical interactions with host proteins [4].

Lenacapavir (GS-6207) is a capsid inhibitor that binds to a pocket formed by two adjacent capsid subunits [5]. It interferes with the dynamics of the assembly and disassembly of the core macromolecule, thereby inhibiting both the late and early stages of HIV-1 replication. The lenacapavir-binding site is also used by two host factors, CPSF6 and NUP153, which are essential for the import of the capsid and its cargo into the nucleus. 

With the recent approval of lenacapavir, HIV-1 capsid has emerged as the newest target of antiretroviral (ARV) therapy. Lenacapavir is the most potent inhibitory ARV in vitro and has demonstrated virologic efficacy in highly ARV-treatment (ART)-experienced patients [5,6]. However, lenacapavir has consistently been selected for drug-resistance mutations when administered without a sufficient number of additional active ARVs to highly ART-experienced patients [6]. Capsid sequencing to identify mutations associated with lenacapavir resistance will, therefore, become necessary for the management of patients with persistent viremia or virological rebound while receiving lenacapavir. 

To assist in the interpretation of clinical laboratory capsid sequencing, we analyzed HIV-1 group M capsid sequences from approximately 20,000 individuals and generated a comprehensive profile of which amino acid mutations had been reported at each capsid position. Mutations missing from this profile were classified as unusual mutations, while mutations indicating likely G-to-A hypermutation were classified as signature APOBEC mutations. We characterized the degree of conservation at each capsid position, the distribution of the numbers of unusual mutations and signature APOBEC mutations per sequence, the influence of subtype and cytotoxic T lymphocyte (CTL) selection pressure on capsid variability, and the degree of covariation among capsid residues. 

## 2. Materials and Methods

### 2.1. Sequence Retrieval

We used the HXB2 capsid amino acid sequence to perform a BLAST search of GenBank on 15 December 2022. The retrieved sequences were grouped into submission sets sharing the same “Title” and “Author” fields. As some submission sets lacked associated PubMed IDs, we searched PubMed using the author fields and Google Scholar using the GenBank title to identify linked publications that were not available at the time the sequences were first submitted to GenBank. Submission sets that could not be linked to a PubMed ID by December 2022 were deemed unpublished. We then reviewed each PubMed reference to identify datasets containing group M HIV-1 capsid sequences from ≥20 HIV-1 infected persons with active virus replication. 

When multiple sequences were available from the same person at multiple time points, we selected the earliest sample for analysis. When multiple clones were available from a sample, we created a consensus sequence from the clones. We included only those sequences encompassing two-thirds or more of the capsid gene. Studies of proviral DNA sequences from persons with virological suppression and studies of HIV-1 quasispecies containing more than three clones per person were excluded. 

### 2.2. Creating a Consensus Sequence

We aligned each of the group M capsid nucleotide sequences using the Biopython package. We then translated each aligned sequence and numbered it from the start of the capsid protein (i.e., 1 to 231). We submitted each of the capsid sequences to the COMET program for subtyping [7] and created consensus amino acid sequences for the following eight main subtypes: A, B, C, D, F, G, CRF01_AE, and CRF02_AG. We created a group M consensus sequence by generating a consensus of the eight main subtypes. For four positions at which four subtypes contained one amino acid and four subtypes contained a different amino acid, we used the subtype C consensus amino acid because subtype C is the most prevalent global subtype. Mutations were defined as amino acid differences from this group M consensus sequence. 

### 2.3. Sequence Quality Control

We created four lists of mutations to assist in performing sequence quality control: (1) a list of mutations strongly suggestive of APOBEC-mediated G-to-A hypermutation, which we called signature APOBEC mutations; (2) a list of usual mutations defined as having a global prevalence ≥0.1%; (3) a list of mutations associated with reduced lenacapavir susceptibility: L56I, M66I, Q67H, K70N, N74D/S, A105E, and T107N [5,8] and (4) a list of unusual mutations defined as mutations that were neither usual, nor signature APOBEC mutations, and were not associated with reduced lenacapavir susceptibility.

To identify signature APOBEC mutations, we combined the sequences in our dataset with the sequences defined by the Los Alamos National Laboratory (LANL) HIV Sequence Database as having APOBEC-mediated G-to-A hypermutation [9]. Signature APOBEC mutations were defined as mutations arising in an APOBEC dinucleotide context (GG→AG for APOBEC3G and GA→AA for APOBEC3F) that were 10 times more frequent in the set of hypermutated sequences than in the remaining sequences in the dataset. This 10-fold cut-off identified 75 mutations with a prevalence < 0.1% in the overall dataset and 19 mutations with a prevalence between 0.1% and 0.3% in the overall dataset.

We used an expectation-maximization procedure to identify sequences containing unexpectedly high numbers of signature APOBEC mutations and sequences containing unusual mutations [10]. We excluded both types of sequences from further analyses. 

### 2.4. Inter- and Intra-Subtype Variation 

For the eight main subtypes, we determined the median inter- and intra-subtype pairwise uncorrected nucleotide and amino acid distances. To determine the impact of the subtype on each amino acid position, we computed the Chi-Square statistic for the contingency matrix containing the number of amino acids in each of the eight main subtypes. This Chi-Square statistic is a measure of the extent to which different subtypes have different amino acids at the same position. 

### 2.5. Amino Acid Profile

Following the quality control steps outlined above, we determined the proportion of each amino acid at each capsid position. The extent of variability at each position was calculated using Shannon entropy. Each amino acid variant in the profile was also characterized according to its evolutionary and hence biochemical relatedness to the position’s consensus amino acid using the BLOSUM62 amino acid similarity matrix. 

HLA-associated positions were defined as capsid positions containing amino acids that were significantly correlated in a phylogenetic context with an HLA type in nine published studies [11,12,13,14,15,16,17,18,19]. These positions were considered likely to be targeted by cytotoxic T lymphocytes (CTLs). For each capsid position, the strength of its association with CTL pressure was defined by the number of studies reporting that the position was associated with an HLA type. We analyzed HLA-associated positions rather than specific CTL epitopes because CTL escape can result from mutations upstream, downstream, and within an epitope [20].

Spearman coefficients were calculated to determine the strength of association between the HIV-1 subtype (using the subtype Chi-Square statistic), CTL pressure (using the number of studies reporting that the position was associated with an HLA type), and sequence variability (Shannon entropy). For this analysis, we analyzed only positions at which one or more mutations had a prevalence ≥1.0% because including positions with less common mutations (e.g., between 0.1% and 1.0%) would artifactually increase the correlation between subtype, CTL pressure, and entropy because it would add a large number of positions containing very low values for all three variables. 

Non-parametric rank-based regression was performed using the Rfit program [21] to independently determine the effects of subtype and CTL pressure on Shannon entropy. By using rank-based coefficients, Rfit assessed the impact of both the subtype Chi-Square statistic and the number of studies reporting that a position was associated with an HLA type even though the scales of these two variables were very different.

### 2.6. Correlation Analysis

To determine whether capsid mutations at different positions had co-evolved we used the HyPhy package to reconstruct the substitution history of the capsid by a maximum likelihood-based phylogenetic method and analyzed the joint distribution of substitution events using a Bayesian graphical model [22]. We identified all pairs of positions that contained mutations in five or more sequences and for which the posterior probability that mutations at the positions were conditionally dependent was greater than 0.99. Once two positions were found to be correlated, we performed Chi-Square tests for each of the pairs of non-consensus amino acids with a prevalence ≥1% at a position to determine which specific pairs of amino acids contributed the most to positional co-evolution.

To explore possible structural explanations for mutational covariation, we plotted the most strongly correlated positions using PyMol and the PDB structure 3H4E which modeled intra-hexameric contacts at 2.7 angstroms resolution [23]. We used 3H4E to calculate the inter-atomic distance between the closest atoms in each pair of amino acids both within each capsid monomer and between adjacent capsid monomers in the same hexamer using Biopython. For 3H4E, distances that included residues at position 220 or greater could not be calculated because they were not included in the PDB file. We used the PDB structure 3J34 which modeled inter-hexameric contacts at 8.6 angstroms resolution [24] to calculate inter-hexameric distances, distances less than 10 angstroms were noted. We also examined two structures of immature capsid CTD: 5I4T [25] and 5L93 [26] for which the resolutions were 3.6 and 3.9 angstroms, respectively.

## 3. Results

### 3.1. References, Patients, and Sequences

The BLAST search returned 215 submission sets containing group M capsid sequences from ≥ 20 persons. We excluded 35 submission sets obtained from studies of viruses from persons without active virus replication and from studies of virus quasispecies for which there were more than three sequences per person or for which the number of persons undergoing sequencing could not be determined. In the remaining 180 publications, there were 24,048 group M HIV-1 capsid sequences from 21,843 persons. Of these, 21,302 (88.6%) encompassed at least two-thirds of capsid positions. 9735 (44.6%) persons also had sequences of one or more *pol* genes including 8511 protease genes, 7510 RT genes, and 5803 integrase genes.

### 3.2. Sequence Quality Control

**Figure 1:**
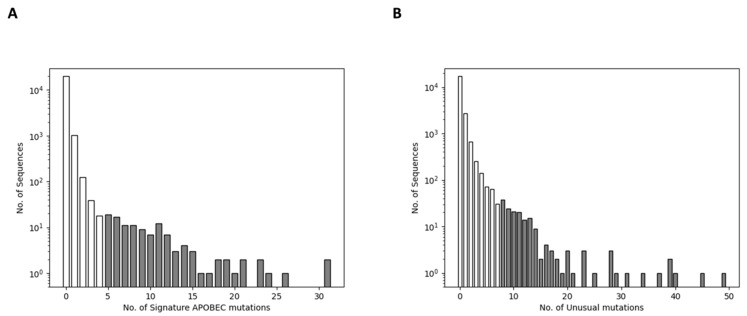
The distribution of the number of signature APOBEC mutations per sequence (**A**) and the number of unusual mutations (defined as having a global prevalence <0.1%; (**B**)) per sequence in the approximately 21,000 published HIV-1 Group M capsid sequences from individuals with active virus replication. Sequences containing ≥5 signature APOBEC mutations or ≥8 unusual mutations, indicated by grey histograms, were considered to belong to a distribution of sequences with an increased risk of sequence artifact by expectation maximization.

We examined the distribution of the number of signature APOBEC mutations per sequence among the 21,302 one-per-person sequences and identified 19,991 sequences containing no signature APOBEC mutations, 1011 containing one such mutation, 125 containing two such mutations, 39 containing three such mutations, 18 containing four such mutations, and 118 containing five or more such mutations (Figure 1A). The presence of five or more signature APOBEC mutations was associated with a false discovery rate of less than 0.1% suggesting that the sequences with this many signature APOBEC mutations have a high likelihood of having undergone APOBEC-mediated G-to-A hypermutation. We, therefore, removed the 118 sequences containing five or more signature APOBEC mutations from further analyses. 

We examined the distribution of the number of unusual mutations (e.g., having a global prevalence <0.1%) per sequence among the 21,184 non-hypermutated one-per-person sequences: 17,067 had no unusual mutations, 2727 sequences had one unusual mutation, 662 had two unusual mutations, 251 had three unusual mutations, 305 had four to seven unusual mutations, and 172 had eight or more unusual mutations (Figure 1B). The presence of eight or more unusual mutations was associated with a false discovery rate of less than 0.1% suggesting that the sequences with this many unusual mutations may have had a quality control issue. We, therefore, removed the 172 sequences containing eight or more unusual mutations from further analysis.

Of the 21,012 sequences passing quality control, 27% were assigned to subtype B, 23% to subtype C, 18% to subtype A, 14% to CRF01_AE, 8.0% to D, 1.9% to subtype F, 1.4% to subtype G, 0.4% to CRF02_AG, 4.6% to other CRFs, 1.2% to URFs, and 0.2% to subtypes H, K, J, and L. Table 1 summarizes the subtypes, sequencing methods, and ARV treatment (ART) histories associated with each of the sequences that passed quality control. The sequencing methods included direct PCR dideoxy terminator sequencing (19,281 persons, 91.8%), single or consensus of molecular clonal sequencing (1211 persons; 5.8%), single or consensus of single genome sequences (420, 2.0%), or consensus of NGS (100, 0.5%). 

### 3.3. Inter- and Intra-Subtype Variability

**Figure 2:**
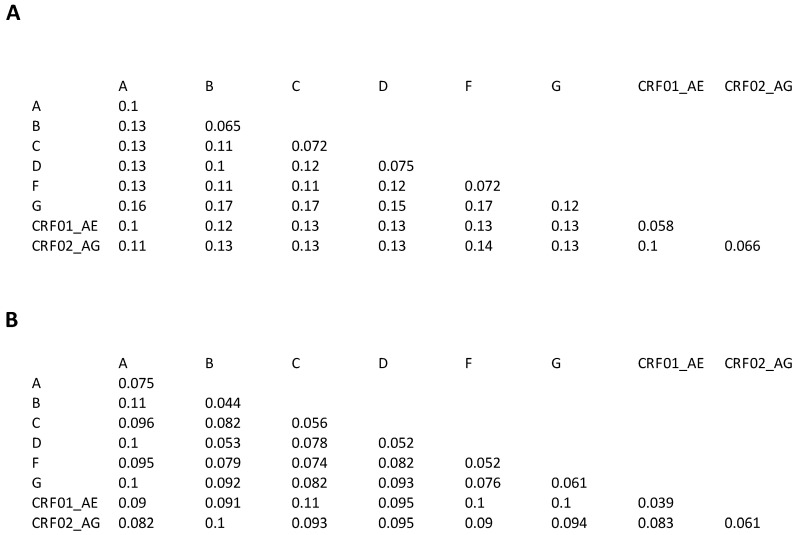
The median intra-subtype and inter-subtype nucleotide (**A**) and amino acid (**B**) distance between capsid sequences belonging to eight major subtypes.

Figure 2A shows the median inter- and intra-subtype pairwise capsid nucleotide distances. Figure 2B shows the median inter- and intra-subtype pairwise amino acid distances. The median inter-subtype nucleotide distance was 0.13 (range: 0.1–0.17) and the median intra-subtype amino acid distance was 0.072 (range: 0.058–0.12). The smallest inter-subtype nucleotide distances were between subtypes B and D and between subtypes CRF01_AE and CRF02_AG. The highest intra-subtype nucleotide distances were for subtype A and the lowest for CRF01_AE. The median inter-subtype amino acid distance was 0.092 (range: 0.052–0.11) and the median intra-subtype amino acid distance was 0.054 (range: 0.039–0.075).

### 3.4. Capsid Amino Acid Profile

**Figure 3:**
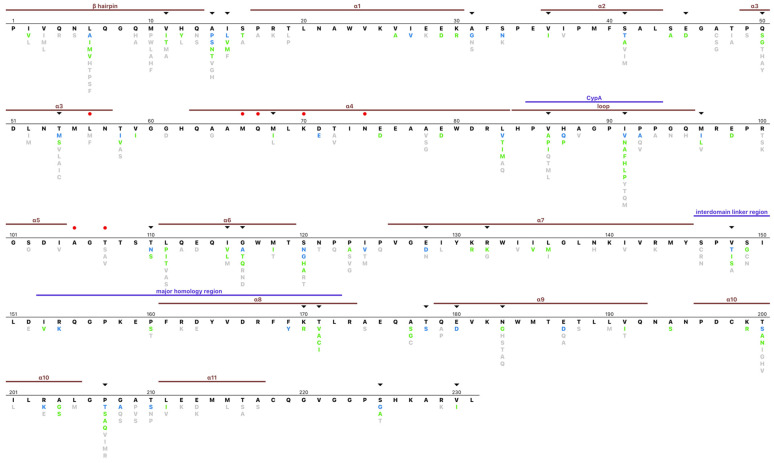
HIV-1 capsid Group M amino acid profile. The consensus sequence is indicated in black. Non-consensus amino acids with a prevalence ≥ 10%, 1.0–10%, and 0.1–1.0% are indicated in blue, green, and grey, respectively. Capsid secondary structural elements and motifs are indicated above the consensus sequence. The red dots indicated positions at which lenacapavir-resistance mutations have been reported. The black triangles indicated positions associated with an HLA-associated polymorphism in ≥ 4 publications.

Figure 3 depicts an amino acid profile for the complete set of group M sequences. In total, 106 (45.9%) positions were completely conserved in that they had no mutations with a global prevalence ≥0.1% (i.e., usual mutations), while an additional 56 (24.2%) positions had only conservative mutations defined as mutations with a prevalence ≥0.1% and a positive BLOSUM62 score). One, two, three, or more than three different mutations (including conservative mutations) were present at 61 (26%), 26 (11%), 18 (7.8%), and 21 (9.1%) positions. At 33 positions, one or more subtypes had a consensus amino acid that differed from the overall group M consensus. Of note, just 70 positions had mutations with a global prevalence ≥1.0%.

The mean biochemical relatedness of each amino acid to the consensus residue as judged by the BLOSUM62 matrix was +0.85, +0.65, and −0.051 for amino acids having a prevalence ≥ 10%, 1.0–9.9%, and 0.1–0.9%. Seventeen positions had an entropy value ≥ 1.0 including positions 6, 14, 15, 41, 54, 58, 83, 91, 92, 96, 116, 120, 128, 148, 200, 207, and 225. Twenty positions had an entropy value between 0.5 and 1.0.

None of the previously reported lenacapavir-resistance mutations (L56I, M66I, Q67H, K70N/H/R, N74D, A105E, and T107N) had a prevalence ≥0.1%. However, uncommon variants were reported at positions 56 and 107 (Supplementary Figure S1). At position 56, L56M and L56F were reported in 0.16% and 0.10%, respectively. L56M occurred primarily in subtypes A and C viruses while L56F occurred primarily in subtype C viruses. At position 107, T107S occurred in 1.0% of sequences while T107A and T107V each occurred in 0.3% of sequences. There was no obvious association of T107 mutations with any particular subtype. An additional 20 positions have been reported to be within 4.5 angstroms of lenacapavir or to interact with lenacapavir including positions 37, 38, 41, 50, 53, 54, 57, 59, 63, 69, 73, 106, 130, 135, 169, 172, 173, 179, 182, and 183 [27]. Eleven of these positions were completely conserved, three had conservative mutations, while positions 31, 41, 50, 54, 179, and 183 contained one or more non-conservative mutations.

Figure 3 depicts several additional noteworthy conserved positions including (1) G89 and P90, which bind to cyclophilin A which promotes peptide bond isomerization [1,4]; (2) K158 and K227, which bind to a negatively charged metabolite, inositol hexakisphosphate (IP6), during capsid assembly [4]; (3) positions that comprise the CTD-end dimer interface, which include L151, E175, V181, K182, W184, M185, L189, and L190 (at position 190, the conservative mutation M occurs in 0.6%) [28]; and (4) the major homology region (MHR; positions 153–172). Other than positions 160 and 171, 12 MHR positions were completely conserved, while six had conservative mutations.

The distribution of capsid mutations in the 323 samples obtained from protease inhibitor (PI)-experienced individuals was similar to the overall distribution of capsid mutations. Seven mutations with a prevalence ≥ 1.0% in PI-experienced individuals occurred 3–8 times more frequently than among the complete set of sequences: I91P (4.8% of PI-experienced individuals), T107S (4.0%), M96V (3.1%), T107A (2.5%), N121T (2.2%), P124T (1.9%), and L205M (1.2%).

### 3.5. HLA-Associated Polymorphisms and Subtype Variability

Seventy-six positions were found to have an HLA-associated polymorphism including 16 positions reported in five to seven studies, 26 positions reported in three to four studies, and 34 positions reported in one to two studies. Among the 39 positions with three or more variants, 33 were reported to contain an HLA-associated polymorphism. 

Entropy was significantly correlated with both subtypes (Spearman rho = 0.83; *p* < 1 × 10^−9^) and by the number of times a position was found to be an HLA-associated polymorphism (Spearman rho = 0.43; *p* = 0.0002). There was no significant association between subtype and HLA (Spearman rho = 0.21; *p* = 0.08) (Supplementary Figure S2A). In the Rfit non-parametric rank-based regression model, both subtype and, to a lesser extent, the number of times that a position was reported to be associated with an HLA type were independently predictive of entropy (Supplementary Figure S2B). 

**Figure 4:**
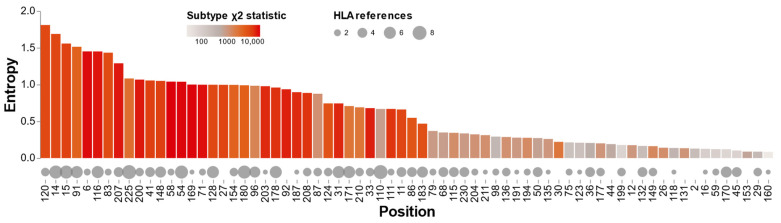
Correlation between Shannon’s entropy, the logarithm of the subtype Chi-Square statistic, and the number of studies reporting that a position was associated with an HLA-associated polymorphism among the 70 capsid positions containing one or more mutation with a prevalence ≥1.0%.

Figure 4 shows the entropy, natural log of the subtype Chi-Square statistic, and a number of publications describing an HLA association for each of the 70 positions containing one or more mutations with a global prevalence ≥1.0%. This figure indicates that for certain positions the association with an HLA type and presumably CTL escape may be a particularly important contributor to entropy. For example, T110N, which occurs at the third position of the TW10 Gag epitope and dominates the CTL response in acute infection in HLA-B57 individuals, was reported in eight publications while its subtype Chi-Square statistic was relatively low [29]. Figure 4 also indicates that there are several positions with low entropy at which several publications reported an association between an HLA type and amino acid variation at the position. 

### 3.6. Correlated Positions

**Figure 5:**
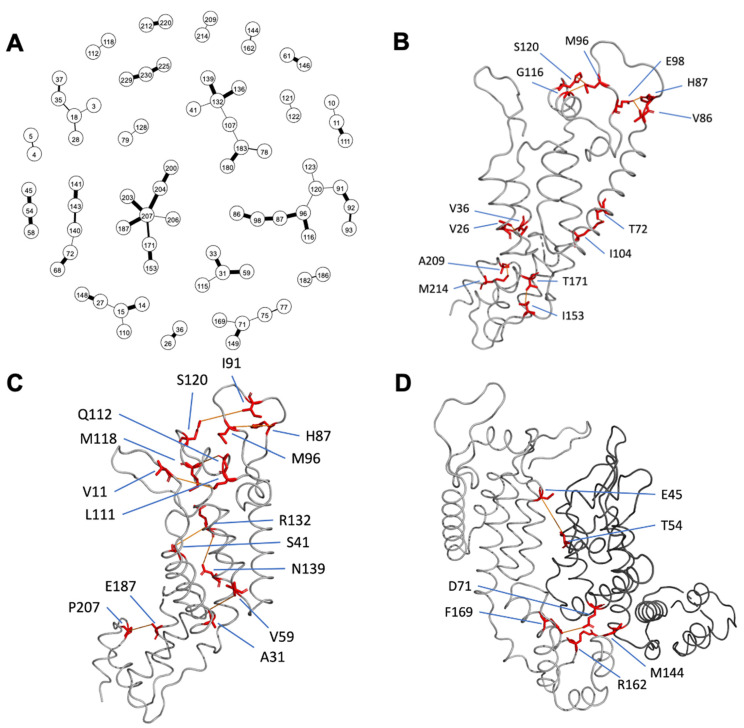
Correlation of amino acid positions. (**A**) Graphical network diagram of all significantly correlated positions. The edge thickness is proportional to the probability of interaction. (**B**) Correlated positions that were ≥5 positions apart and were within 5 angstroms in the same monomer, residues were linked in red lines by closest atoms. (**C**) Correlated positions that were ≥5 positions apart and between 5 and 10 angstroms in the same monomer. (**D**) Correlated positions which that were ≥5 positions apart and within 10 angstroms between adjacent monomers in a hexamer.

Mutations at 58 pairs of positions were considered to be highly correlated based on having mutations in ≥5 sequences and a conditional dependence probability ≥ 0.99. These included eight pairs of amino acids that were adjacent to one another in the linear peptide sequence, fifteen pairs that differed by two-to-four positions in the linear sequence, and 35 pairs that differed by ≥5 positions in the linear sequence. Figure 5A shows a graphical network diagram of all significantly co-dependent positions with the edge thickness proportional to the inferred probability of interaction.

Among the 35 correlated pairs of positions that were greater than five positions apart in the linear sequence, inter-atomic distances could be calculated for 34 pairs (i.e., one pair included amino acids above position 220). In the mature 3H4E structure [23], eight pairs of positions had a closest inter-atomic distance of ≤ 5.0 angstroms and eight pairs had an interatomic distance of between 5.0 and 10.0 angstroms in the same monomer (Figure 5B,C). Two pairs had inter-atomic distances of ≤ 5.0 angstroms and one pair had an interatomic distance of between 5.0 and 10.0 angstroms in adjacent monomers (Figure 5D). Fifteen pairs or residues had a distance of > 10 angstroms from each other. 

Of the fifteen distant pairs of positions, four were associated with the same HLA type including positions 15 and 110 which were associated with HLA B*57 and C*06 [11,13,14,15,16,17,18,19], positions 31 and 115 which were associated with HLA B*57 [11,13,14,17,18,19], positions 71 and 149 which were associated with HLA B*52 [16], and positions 171 and 207 which were associated with HLA B*58 and C*03 [13,14,17,18,19]. Among the 35 correlated pairs, 21 pairs of positions had 68 pairs of amino acids which co-occurred more frequently than expected by chance after controlling for multiple comparisons using Holm’s sequential Bonferroni procedure (Supplementary Table S1).

### 3.7. Database and Sequence Interpretation Program

The 180 papers and their associated sequences were added to the Stanford HIV Drug Resistance Database (HIVDB). We reviewed each paper and annotated the sequences with their subtypes, sequencing and cloning methods, and with the ART histories of the individuals from whom the sequences were obtained. Additionally, we included the mutations identified in ten individuals who received lenacapavir in two clinical trials in our database. The capsid sequence query page can be accessed at the following URL: https://hivdb.stanford.edu/cgi-bin/InhibitorsMutations.cgi?Gene=CA (accessed on 22 March 2023). The HIVDB drug resistance interpretation program has been supplemented with a program that analyzes HIV-1 capsid sequences. Upon submission of either FASTA or FASTQ (next-generation sequencing; NGS) files, the program reports the detected mutations, the proportion of reads containing each mutation (for FASTQ files), and whether the mutations are lenacapavir resistance mutations, unusual mutations, or signature APOBEC mutations.

## 4. Discussion

Genotypic resistance testing is important in the selection of ARVs belonging to the four major drug classes: nucleoside RT inhibitors, non-nucleoside RT inhibitors, protease inhibitors, and integrase strand transfer inhibitors. With the recent approval of lenacapavir, capsid sequencing will be required for the management of heavily ART-experienced patients with detectable viremia while receiving lenacapavir. Our analysis provides information essential for the quality control of capsid sequencing, as the identification of many unusual mutations in a sequence suggests that some of the mutations may represent sequence artifacts or, less likely, a novel variant. Identifying sequences with an unexpectedly high number of unusual or signature APOBEC mutations is particularly useful for NGS to help users select an appropriate mutation-detection threshold because an excess of unusual or signature APOBEC mutations at a low mutation-detection threshold indicates that the threshold should be raised [30].

HIV-1 group M capsid sequences are highly conserved. Nearly 70% of positions were completely conserved in that they contained no mutations with a prevalence ≥ 0.1% (i.e., had no usual mutations) or had only conservative mutations (usual mutations with a positive BLOSUM62 score). This level of conservation reflects the requirement for each capsid monomer to interact through distinct interfaces with at least three other monomers and with several host proteins [31]. Additionally, the capsid may be the most important target of cell-mediated immunity because large numbers of capsid monomers either comprise the HIV-1 core or are packaged within the core making peptides derived from the capsid the earliest presented to the immune system following initial infection [32,33]. The combination of high levels of conservation and the need to escape cell-mediated immunity may explain why just 37 (16.0%) positions have entropy levels ≥ 0.5. 

Most of the variation at highly entropic positions appears to be explained by both subtype variability and CTL selection pressure. Subtype variability is likely caused by founder effects as HIV-1 evolves in different regions and populations. Capsid variability correlates most strongly with subtype variability indicated by the Chi-Square statistic at each capsid position. The distribution of variants in our dataset was influenced by the frequency of sequencing and reporting of viruses belonging to different subtypes. Subtype B viruses, which globally account for 12% of viruses [34], accounted for 27% of viruses in our dataset.

Lenacapavir inhibits HIV-1 by stabilizing and preventing the carefully choreographed disassembly of the capsid that occurs following cell entry [35]. Lenacapavir binds two adjacent capsid subunits in a pocket that is also recognized by two cellular cofactors, CPSF6 and Nup153, that mediate viral nuclear import. Mutations at several positions have been selected in vitro during lenacapavir passage experiments and/or in 10 patients receiving lenacapavir, including L56I, M66I, Q67H, K70N/H/R, N74D, A105E, and T107N [5,6,36]. With the exception of positions 56 and 107, where L56M/F and T107S/A/V can occur in the absence of selective drug pressure, the remaining five positions are completely conserved in circulating isolates.

The two most frequently occurring lenacapavir-resistance mutations in patients have been M66I and Q67H [6,8]. M66I has been reported to confer >1000-fold reduced lenacapavir susceptibility and a low replication capacity of between 2% and 6% [8]. It has been reported in six of 10 patients with emergent resistance in clinical trials usually in combination with one or more additional lenacapavir-resistance mutations. Q67H has been reported to confer approximately five-fold reduced lenacapavir susceptibility and to have a replication capacity of between 58% and 100% [8]. It has been reported in five of 10 patients with emergent lenacapavir resistance usually in combination with M66I and/or K70R.

Our analysis of variability at lenacapavir-resistance positions is consistent with two previous studies that examined 10,862 and 23,671 one-per-person sequences downloaded from the LANL database in 2013 and 2021, respectively [37,38]. It is also consistent with a study of 1500 unpublished sequences from France that included 500 sequences from ART-naïve individuals, 500 from ART-experienced PI-naïve individuals, and 500 obtained from PI-experienced individuals [39]. However, it differs from a study of 2031 sequences from ART-naïve individuals in the LANL database, which reported several unusual mutations at lenacapavir resistance positions, including M66C (4.7% of sequences), Q67K (3.8%), N74R (2.8%), and T107L (4.0%) [40]. We were unable to identify these mutations in our BLAST search or in an additional LANL search. 

In contrast to previous studies, our analyses were based on sequences obtained from a BLAST search of GenBank. This allowed us to populate HIVDB with capsid sequences according to the reference in which they were reported and to annotate them by subtype, ART history, and method of sequencing and cloning. We focused our efforts on those studies reporting sequences from twenty or more individuals and did not analyze sequences that were reported from virologically suppressed individuals as the proviral DNA reservoir is dramatically enriched for viruses with viral genomic errors and replication-incompetent sequences [41]. 

In conclusion, knowledge of the distribution of unusual and signature APOBEC mutations will be essential for sequence quality control. This is particularly important for sequences obtained using NGS technologies because the presence of high numbers of signature APOBEC mutations and unusual mutations at a low mutation detection threshold suggests that the threshold should be raised. In addition, comparing capsid sequences from lenacapavir-treated individuals with previously published sequences from lenacapavir-naïve individuals will enable the discovery of additional mutations potentially associated with lenacapavir therapy.

## Figures and Tables

**Table 1:** Sequence characteristics.

Characteristic	Sequences (n = 21,012)
**Subtype**	n (%)
A	3841 (18)
B	5716 (27)
C	4806 (23)
D	1668 (8.0)
F	399 (1.9)
G	291 (1.4)
CRF01_AE	2967 (14)
CRF02_AG	84 (0.4)
Other CRF	956 (4.6)
URF	249 (1.2)
H	21 (0.10)
J	6 (0.03)
K	7 (0.03)
L	1 (0.005)
**Treatment histories**	
None	19,199 (91.4)
RTI	442 (2.1)
PI	323 (1.5)
Unknown	1371 (6.5)
**Sequencing method**	
PCR dideoxy terminator sequencing	19,281 (91.8)
Single or consensus of molecular clonal sequencing	1211 (5.8)
Single or consensus of single genome sequences	420 (2.0)
Consensus of NGS	100 (0.5)

## Data Availability

The raw capsid sequence data can be found on https://hivdb.stanford.edu/cgi-bin/InhibitorsMutations.cgi?Gene=CA (accessed on 22 March 2023). The analysis results can be found on https://github.com/hivdb/hiv-capsid-data (accessed on 22 March 2023).

## Supplementary Materials

The following supporting information can be downloaded at: https://www.mdpi.com/article/10.3390/v15040992/s1, Figure S1: Lenacapavir-resistance positions shown within the context of capsid sequence variability; Table S1: Amino acid pairs which co-occurred more frequently than expected by chance after controlling for multiple comparisons using Holm’s sequential Bonferroni procedure; Figure S2: Correlation between Shannon’s entropy, the logarithm of the subtype Chi-Square statistic, and the number of studies reporting that a position was associated with an HLA-associated polymorphism among the 70 capsid positions containing one or more mutation with a prevalence ≥1.0% with Spearman’s rank correlation coefficient (A), or Rfit non-parametric rank-based regression model (B).
