# Supplementary material for: HIV-1 Group M Capsid Amino Acid Variability: Implications for Sequence Quality Control of Genotypic Resistance Testing
Source: Viruses. 2023 Apr 18;15(4):992. doi: 10.3390/v15040992 (PMC10143361; doi:10.3390/v15040992)
Supplement: Supplementary file 1 [file viruses-15-00992-s001.zip › Figure S2.pdf]

A

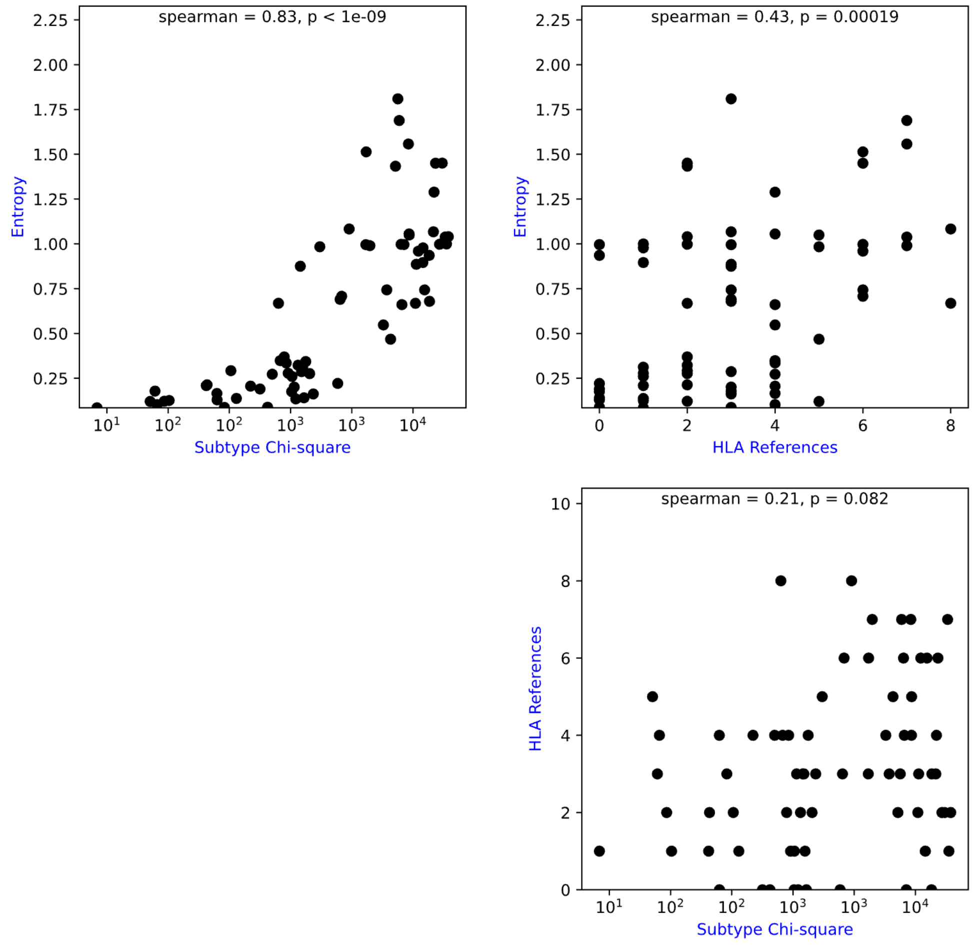

B

```
## Call:
## rfit.default(formula = Entropy ~ subtype + HLA, data = df)
##
## Coefficients:
##                Estimate Std. Error t.value   p.value
## (Intercept) 8.0382e-02 5.0127e-02  1.6036    0.1135
## subtype     5.5344e-05 4.8157e-06 11.4923 < 2.2e-16 ***
## HLA         6.8826e-02 1.2855e-02  5.3540 1.131e-06 ***
## ---
## Signif. codes:  0 '***' 0.001 '**' 0.01 '*' 0.05 '.' 0.1 ' ' 1
##
## Multiple R-squared (Robust): 0.6394636
## Reduction in Dispersion Test: 59.41712 p-value: 0
```
