# Supplementary figures and images for: HIV-1 Group M Capsid Amino Acid Variability: Implications for Sequence Quality Control of Genotypic Resistance Testing
Source: Viruses. 2023 Apr 18;15(4):992. doi: 10.3390/v15040992 (PMC10143361; doi:10.3390/v15040992)

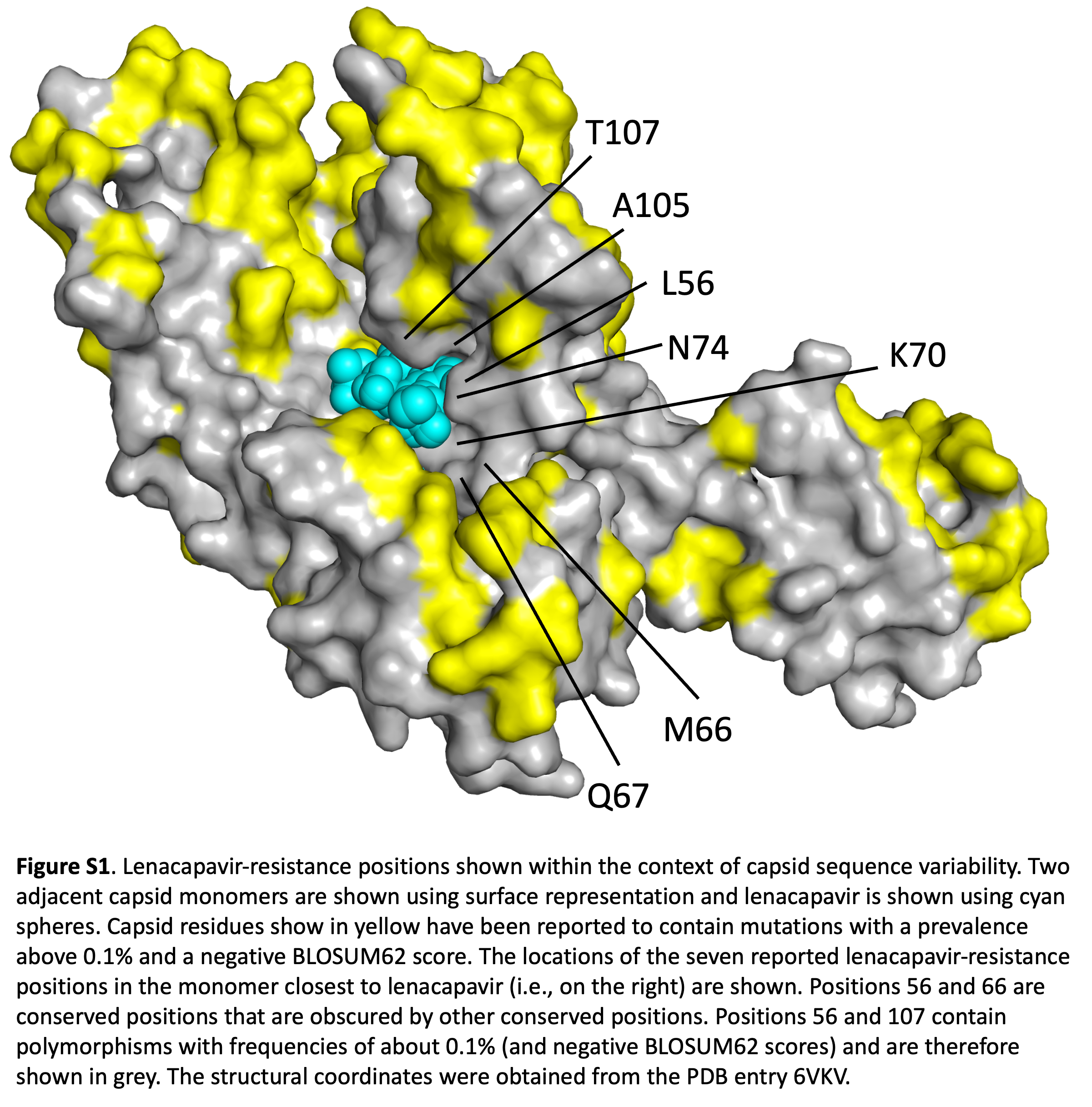

Supplement: Supplementary file 1 [file viruses-15-00992-s001.zip › Figure S1.png]
